# Diagnostic model based on bioinformatics and machine learning to distinguish Kawasaki disease using multiple datasets

**DOI:** 10.1186/s12887-022-03557-y

**Published:** 2022-08-30

**Authors:** Mengyi Zhang, Bocuo Ke, Huichuan Zhuo, Binhan Guo

**Affiliations:** 1 Department of Laboratory Medicine, West China Second University Hospital, Sichuan University, No. 20, Section 3, Renmin South Road, Chengdu, 610041, PR Sichuan Province China; 2 Key Laboratory of Birth Defects and Related Diseases of Women and Children (Sichuan University), Ministry of Education, Chengdu, China

**Keywords:** Kawasaki disease, Diagnostic model, Children, Machine learning

## Abstract

**Background:**

Kawasaki disease (KD), characterized by systemic vasculitis, is the leading cause of acquired heart disease in children. Herein, we developed a diagnostic model, with some prognosis ability, to help distinguish children with KD.

**Methods:**

Gene expression datasets were downloaded from Gene Expression Omnibus (GEO), and gene sets with a potential pathogenic mechanism in KD were identified using differential expressed gene (DEG) screening, pathway enrichment analysis, random forest (RF) screening, and artificial neural network (ANN) construction.

**Results:**

We extracted 2,017 DEGs (1,130 with upregulated and 887 with downregulated expression) from GEO. The Gene Ontology (GO) and Kyoto Encyclopedia of Genes and Genomes (KEGG) analyses showed that the DEGs were significantly enriched in innate/adaptive immune response-related processes. Subsequently, the results of weighted gene co-expression network analysis and DEG screening were combined and, using RF and ANN, a model with eight genes (*VPS9D1*, *CACNA1E*, *SH3GLB1*, *RAB32*, *ADM*, *GYG1*, *PGS1*, and *HIST2H2AC*) was constructed. Classification results of the new model for KD diagnosis showed excellent performance for different datasets, including those of patients with KD, convalescents, and healthy individuals, with area under the curve values of 1, 0.945, and 0.95, respectively.

**Conclusions:**

We used machine learning methods to construct and validate a diagnostic model using multiple bioinformatic datasets, and identified molecules expected to serve as new biomarkers for or therapeutic targets in KD.

**Supplementary Information:**

The online version contains supplementary material available at 10.1186/s12887-022-03557-y.

## Background

Systemic vasculitis is the main pathological feature of Kawasaki disease (KD), which frequently occurs in children between 6 months and 5 years of age. The most prominent comorbidity of KD is coronary artery lesions (CALs), causing coronary artery aneurysm (CAA) expansion, stenosis, thrombosis, myocardial infarction, and sudden death [1–3]. Although early standard treatment can considerably reduce complications in acute KD, 5% of children with KD still present with CALs [4]. Therefore, KD is considered to be a form of childhood “coronary heart disease” related to adult coronary heart disease [4, 5]. Since its discovery, KD has mainly been associated with heart disease in children in developed countries [6].

According to the latest diagnostic guidelines, KD is primarily defined by the following clinical features: 1) fever, 2) diffuse oropharyngeal mucosa hyperemia, 3) rash, 4) redness and swelling of the hands and feet in the acute phase and peeling of the nails during the recovery phase, 5) non-purulent cervical lymphadenopathy, and 6) conjunctival hyperemia [7]. A comprehensive assessment of the disease is performed based on the above-mentioned clinical symptoms and presence of aberrant coronary arteries (such as dilated arteries). Additionally, corresponding laboratory experiments and imaging examination can help in KD diagnosis. The pathogenesis of KD is considerably related to complex influencing factors, namely infection, genetic susceptibility, and immune response, resulting in notable disease heterogeneity across individuals and difficulty in diagnosis. Several studies have reported the presence of Epstein-Barr virus, coronavirus, and hepatitis virus in either peripheral blood or respiratory secretions of patients with KD [8–11]. However, these reports need further confirmation through experiments owing to the poor replicability. Multiple cytokines in the innate immune system of patients with KD can induce coronary inflammation in response to pathogen invasion [12, 13].

Additionally, the adaptive immune response is considerably activated. Recent studies have shown that both pro-inflammatory and regulatory T cells in the blood play critical roles in regulating the severity and susceptibility to KD [14, 15]. Although many single nucleotide polymorphisms associated with KD are homologous in other inflammatory diseases such as rheumatoid arthritis, ulcerative colitis, and systemic habitual lupus erythematosus, the exact molecular mechanism underlying KD has not been elucidated [16–18].

Numerous microarray/sequencing data of gene expression have been published in public databases such as Gene Expression Omnibus (GEO) during the past few years, and they are being increasingly used in bioinformatics to explore target genes or proteins involved in various diseases. These data are classified as high-dimensional sample data, analyzed using machine learning methods for uncovering patterns to elucidate disease pathogenesis and predict diagnostic markers. In this study, we aimed to develop and validate a diagnostic model based on bioinformatics and machine learning to distinguish patients with KD using multiple datasets. The results of this study will provide new insights for future studies to explore the molecular mechanism underlying KD.

## Methods

### Dataset access and preprocess

GEO was used to retrieve the sequencing and microarray datasets used in our study, from which the datasets of patients with KD, normal controls, and convalescent individuals (GSE73461, GSE68004, GSE63881, GSE73463, and GSE109351) were obtained. Considering the phenotypic differences between individuals with the disease and healthy controls, we selected a subset of these datasets. The GSE73461 dataset contained transcriptional profiles of 78 patients with KD and 55 healthy control samples obtained using genome-wide analysis [19], and it was used for differentially expressed gene (DEG) screening, gene enrichment analysis, and random forest construction. The GSE68004 dataset contained data on 76 patients with KD and 37 healthy control samples and was used to construct and validate the ANN model [20]. Both GSE63881 (171 patients with KD and 170 convalescent samples) [21] and GSE73463 (146 patients with KD and 87 convalescent samples) [19] datasets were used for investigating the molecular mechanisms underlying KD with the ANN model. In addition, the GSE109351 (three samples each of patients with KD, healthy controls, and convalescent samples) dataset was used to validate the expression of genes in the ANN model [22]. All datasets except GSE109351 were created using the GPL10558 Illumina HumanHT-12 V4.0 expression bead chip platform, whereas GSE109351 was created using the GPL17586 Affymetrix Human Transcriptome Array 2.0 platform, and data collation and analysis were conducted using R software (version 4.0.3). Platform annotation information was obtained through GEO, and gene annotation was performed with probes using the “org.Hs.eg.db” R package.

### Screening and enrichment of DEGs

Before screening DEGs, the original data were normalized using the R package “Limma,” which was used to identify DEGs from GSE73461, and fold change > 4 and *p* < 0.05 were used as cut-offs for selection [23]. Thereafter, we used R packages “heatmap” and “ggplot2” to show the DEGs with upregulated and downregulated expression, respectively. The R package “clusterProfiler” was used to analyze the enrichment of gene clusters and classification of biological terms via Gene Ontology (GO) and the Kyoto Encyclopedia of Genes and Genomes (KEGG) [24, 25]. The enrichment results were ranked based on the *p*-value; statistical significance was set at *p* < 0.05. The top 10 significance terms were selected for displaying enriched genes and were visualized using R package “ggplot2.”

### Gene co-expression module construction

The R package “WGCNA” was used to construct gene co-expression modules using the top 5000 genes exhibiting statistically significant median absolute deviation in the GSE73461 dataset. Numbers 2–30 were selected as the preset soft threshold β and filtered using the network topology analysis function “pickSoftThreshold” in R. Thereafter, the best β value was screened based on the visualization results of “Scale independence” and “Mean connectivity.” Subsequently, the proximity matrix was constructed using the soft-threshold power value β and transformed into a topological overlap matrix, which was then used to calculate the distance between genes for hierarchical clustering. Moreover, gene modules were generated via dynamic shearing in R and distinguished by color, according to the optimal value β, and the size of genes in module ≥ 30 as the selection criterion. Clinically, the most valuable data for GSE73461 are the groupings by phenotype, namely “Bacterial,” “Control,” “Inflammatory,” “Kawasaki,” “Unknown,” and “Viral.” Therefore, we calculated and screened the obtained gene modules most related to phenotype using the Pearson correlation coefficient test and visualized using “WGCNA.”

### Random forest classification and neural network construction

The R package “randomForest,” generally used to train and predict samples [26], was used to construct classification models of datasets. Before modeling, we randomly sampled the partial data of GSE73461 (contained 78 patients with KD and 55 healthy control samples), divided into a training set and validation set at the ratio of 7:3. The initial variable number for the binary tree in the node was set as system default value, and optimal number of trees was set to 3,000 to construct an initial model. In our study, candidate genes after intersection from DEGs and module genes of WGCNA were entered, and disease-specific genes were chosen according to the screening threshold of mean decrease in Gini and mean decrease in accuracy. Additionally, the five-fold cross-validation method based on machine learning was used to screen suitable candidate genes for constructing random forests to determine the optimal combination of gene numbers. After selection, another dataset, GSE68004, was chosen for ANN model training using the R package “neuralnet” [27]. Eight candidate genes were input, and 5 hidden layers and 2 outputs (KD and healthy control) were set as parameters for constructing a KD model (termed neuralKD).

### Validation of model through machine learning

To further evaluate the detection performance of neuralKD in classification, we used quadratic discriminant analysis (QDA), principal component analysis (PCA), mixed discriminant analysis (MDA), and multiple logistic regression to perform validation with the R packages (including “FactoMineR,” “factoextra,” “mda,” and “MASS”). Before analysis, we randomly sampled the data of GSE68004 (contained 76 patients with KD and 37 healthy control samples), divided into a training set and validation set at the ratio of 8:2. R package "Caret" was used to perform a five-fold cross-validation of AUC, through which the average scores can be determined. Moreover, we visualized the results of candidate genes using the R package “heatmap.” Finally, the R package “pROC” was used to evaluate the performance of the classifier of “neuralKD.”

### Additional data verification

We used two more datasets (GSE63881 and GSE73463) to explore the ability of the model to evaluate the progression of KD. A boxplot was constructed using the expression of eight genes (*VPS9D1*, *CACNA1E*, *SH3GLB1*, *RAB32*, *ADM*, *GYG1*, *PGS1*, and *HIST2H2AC*) in neuralKD between the KD and convalescent groups. Furthermore, the receiver operating characteristic (ROC) curve was analyzed to evaluate the performance of the classifier. In addition, the correlation among genes involved in neuralKD was calculated and visualized using the R package “corrplot.”

## Results

### Workflow

**Fig. 1:**
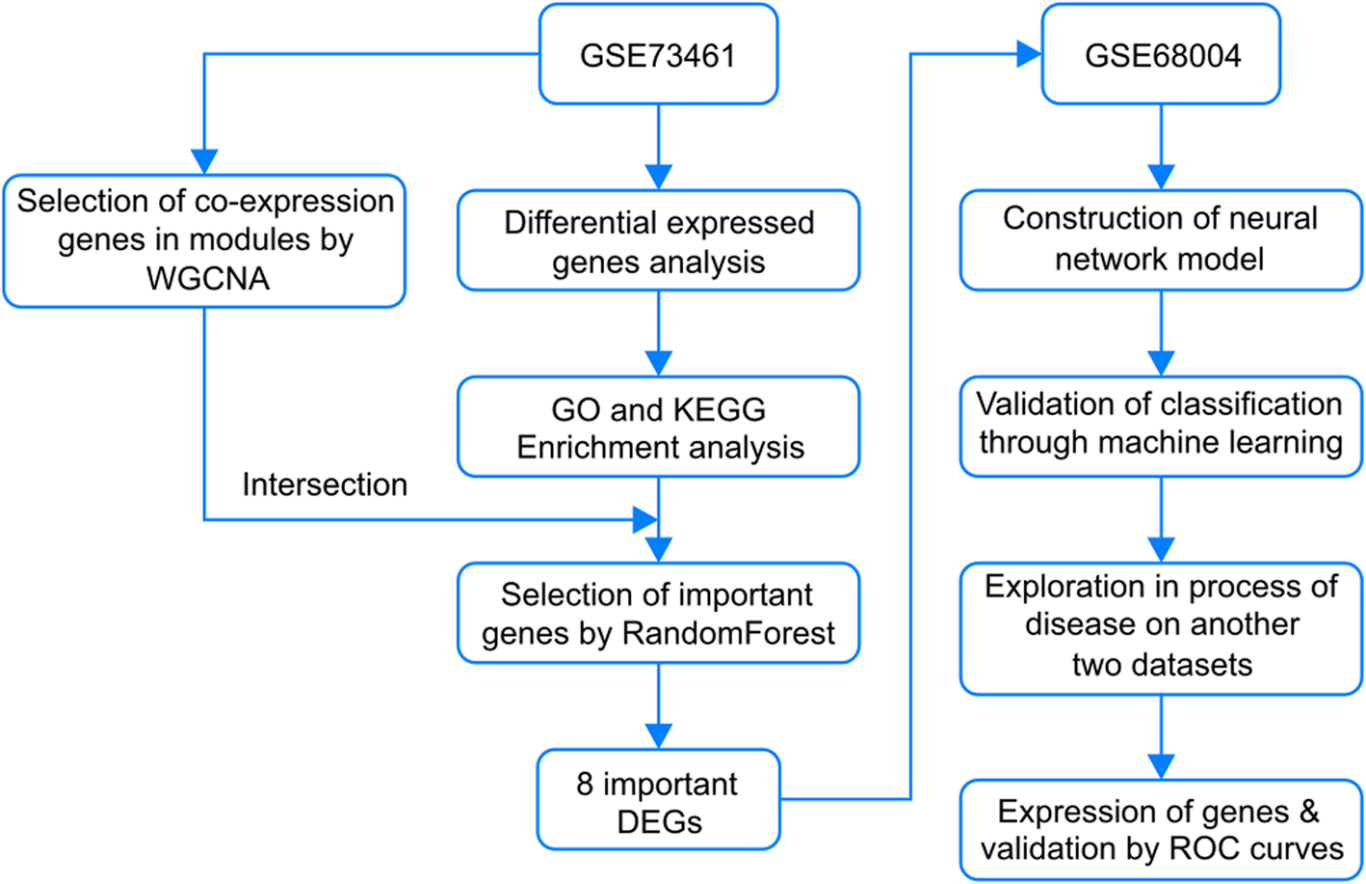
Flow chart of the study, including gene screening and machine learning model construction and validation

Figure 1 displays an outline of the workflow followed in our study.

### Screening of DEGs

**Fig. 2:**
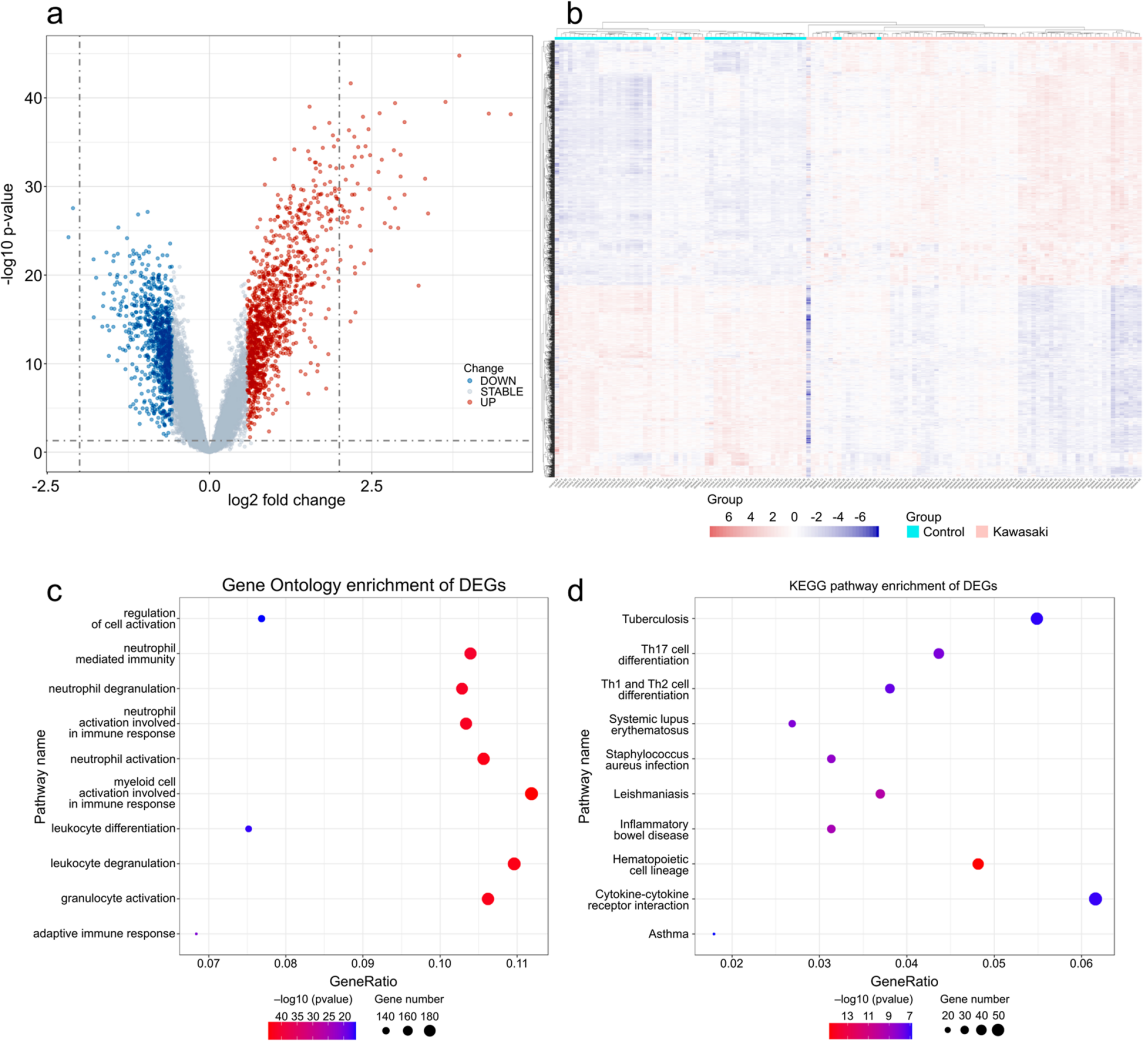
Analyses of DEGs in the GSE73461 dataset. This dataset includes 78 KD and 55 healthy control individuals. **a** Volcano plot of DEGs. **b** Heatmap of DEGs. The correlation between color and fold change of DEG level is displayed in the upper right corner. **c** Top 10 significantly enriched GO terms of DEGs. **d** Top 10 significantly enriched KEGG pathways of DEGs. In (**a**) and (**b**), the genes with upregulated expression are indicated in red, whereas those with downregulated expression are indicated in blue. In (**b**), “Kawasaki” means KD individuals, whereas “control” means healthy control individuals

We extracted 1,130 DEGs with upregulated and 887 with downregulated expression from GSE73461. The results of the DEG analysis are shown in Fig. e 2a and b. The results of the GO and KEGG analyses were conducted from R package "clusterProfiler", which was shown in Fig. 2c and d, respectively. DEGs were significantly enriched in neutrophil-related immune responses, such as “neutrophil mediated immunity” and “neutrophil activation.” The KEGG pathway analysis (*p* < 0.05) suggested that the DEGs were primarily involved in “cytokine–cytokine receptor interaction” and other T cell-related processes such as “Th1 and Th2 cell differentiation.”

### Weighted co-expression network construction and module identification

**Fig. 3:**
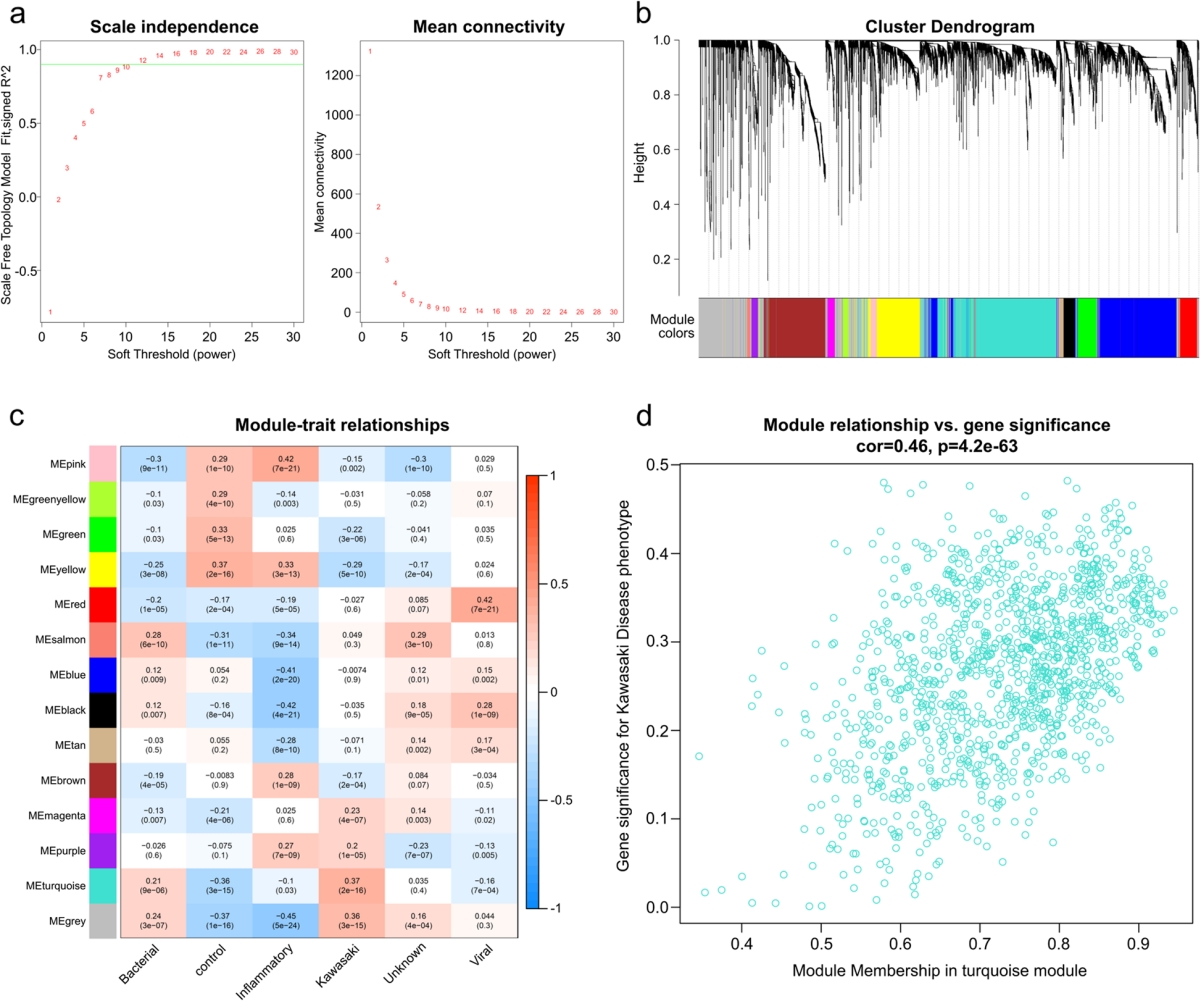
Results of the WGCNA of GSE73461. **a** Network topology analysis of various soft-threshold power. Soft-threshold power value β = 10 was chosen. **b** Cluster dendrogram of the 5,000 genes from GSE73461 was ordered by hierarchical clustering of genes based on the value of dissimilarity. Each branch in the figure represents one gene, and each module is assigned different colors. **c** Heatmap of correlation between gene modules and different phenotypes of GSE73461. Correlation coefficient, along with the *p*-value, is illustrated in parenthesis underneath. The color was coded according to the correlation coefficient (legend at right). **d** Scatter plot of module eigengenes in the turquoise modules in the KD group

For weighted gene co-expression network analysis (WGCNA), we set 30 as the least number of genes in each gene network and 0.9 as the cut height (Fig. 3a). We generated 14 modules when the connectivity between genes in the network satisfied the scale-free network distribution (Fig. 3b). As we had a summary profile (eigengene) for each module, we correlated eigengenes with different phenotypes of GSE73461 and searched for the most significant associations (Fig. 3c). The turquoise module positively correlated with the “Kawasaki” phenotype from group information in GSE73461, compared with other modules. The degree of correlation between the genes in the turquoise module and KD phenotype was illustrated using statistical models (Fig. 3d). Additionally, 818 genes from the turquoise module that possibly act in the molecular mechanism underlying the pathogenesis of KD (called “module genes”) were obtained for further analysis.

### Random forest screening for DEGs and neural network construction

**Fig. 4:**
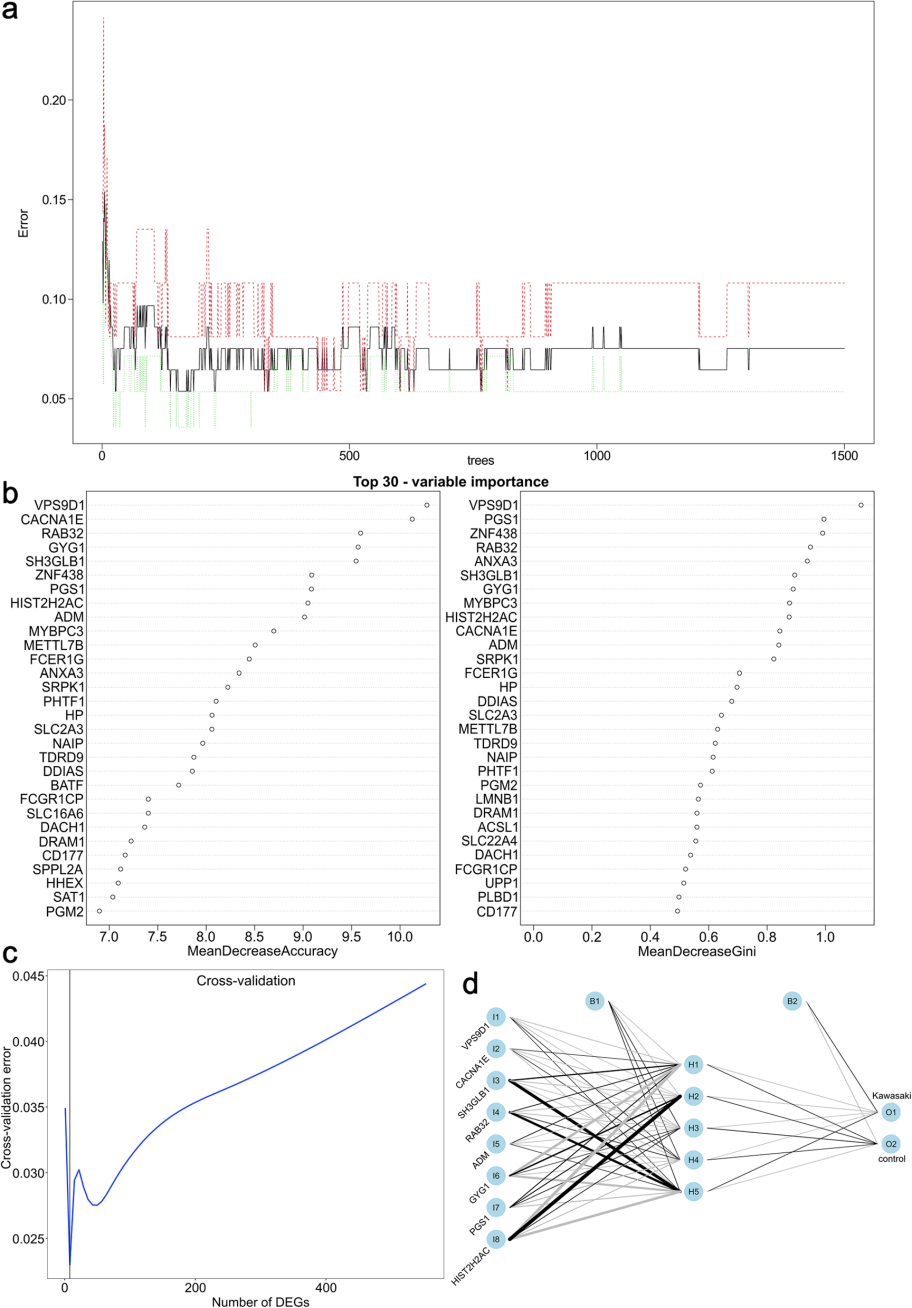
Screening results of Kawasaki disease-related DEGs using a random forest classifier. **a** Influence of the number of decision trees on the error rate. The x-axis is the number of decision trees, and the y-axis is the error rate. **b** Ranking of input variables in the random forest model to classify KD and healthy control samples. All genes are sorted by the value of “MeanDecreaseAccuracy” and “MeanDecreaseGini,” separately. **c** Gene number screening from fivefold cross-validation method in the construction of random forest. **d** Visualization of neural network topology of GSE68004 with 8 input layers, 5 hidden layers, and 2 output layers

To obtain reliable genes that might act as diagnosis markers for KD, we input the candidate 553 genes into the RF classifier after merging the DEGs from GSE73461 with the “module genes” from WGCNA. The best variable number for the binary tree in the node was set as 23, whereas the optimal number of trees in the RF classifier was set to 1,500 to obtain the dimensional importance of all variables (Fig. 4a). The variable importance of the top 30 genes input to the random forest model is shown in Fig. 4b. Eight genes (*VPS9D1*, *CACNA1E*, *SH3GLB1*, *RAB32*, *ADM*, *GYG1*, *PGS1*, and *HIST2H2AC*) were selected for further analysis following evaluation with the cross-validation method in the RF model (Fig. 4c). Thereafter, we used GSE68004 to construct an ANN model with 8 input layers, 5 hidden layers, and 2 output layers to classify the phenotype between disease and normal samples, as shown in Fig. 4d.

### Validation of classification based on machine learning

**Fig. 5:**
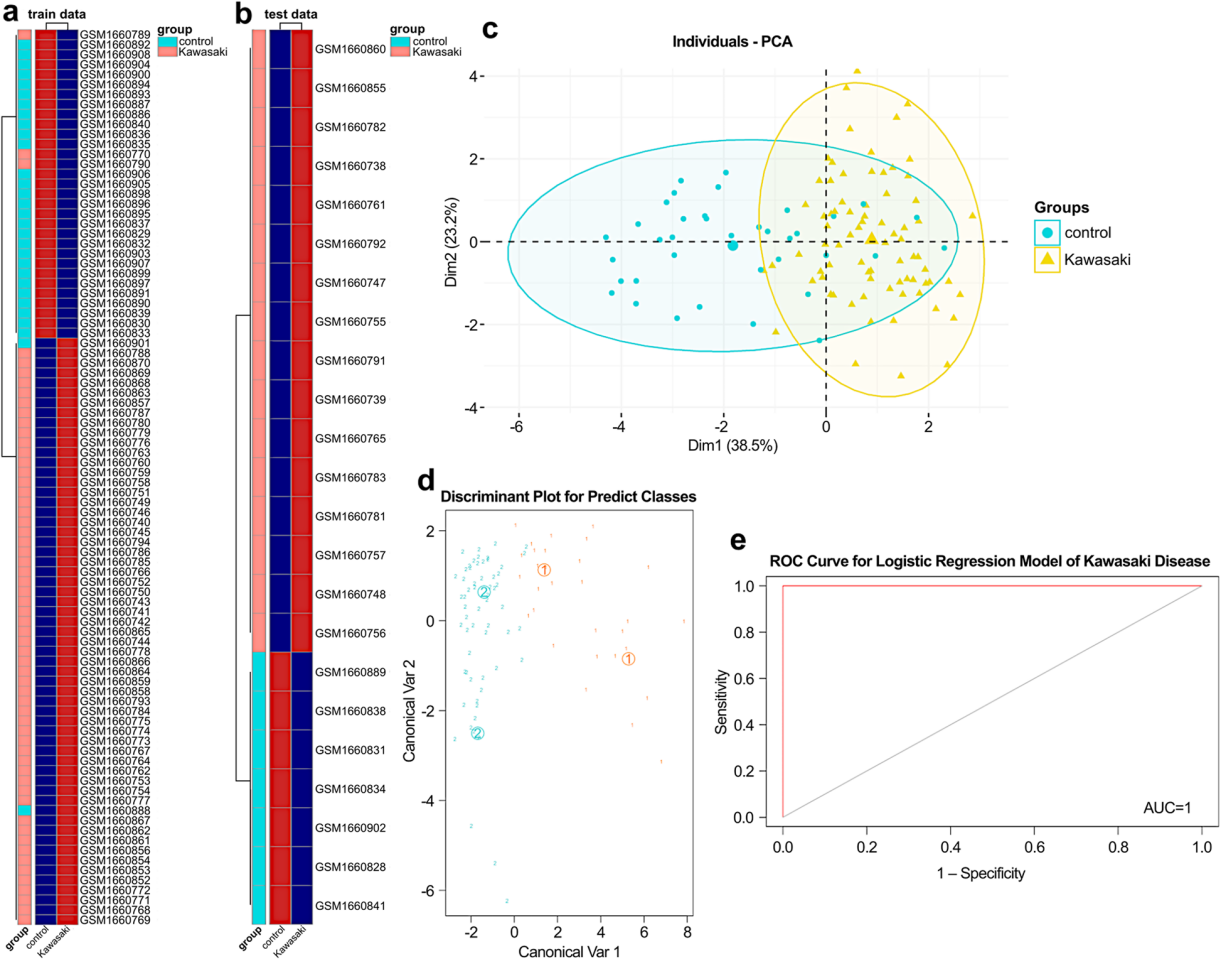
Validation of classification through machine learning. **a** Heatmap for classifying on the GSE68004 training set based on quadratic discriminant analysis algorithm. **b** Heatmap for classifying the GSE68004 validation set based on the QDA algorithm. **c** Classification results based on PCA with the GSE68004 dataset. **d** Classification results based on MDA. **e** Performance evaluation of logistic regression model in GSE68004 by area under the ROC curves and the AUC values (AUC = 1)

To test the classification performance of neuralKD, we used machine learning methods including QDA, MDA, and PCA. The classification obtained using QDA in the training or validation sets was consistent with the actual classification of the samples (Fig. 5a and b). Additionally, the classification results of PCA were flawed, possibly because PCA is an unsupervised algorithm that lacks the label corresponding to the sample, leading to the deviation in classification (Fig. 5c). Moreover, MDA showed significant group differences (Fig. 5d). The area under the ROC curve values were used to evaluate the performance of the logistic regression model in GSE68004, as shown in Fig. 5e. Simultaneously, we comprehensively evaluated the ability of “neuralKD” on other algorithms based on accuracy, F1 score, and AUC (Additional File 1 Table S1). The five-fold cross-validation results were used to confirm the reliability and stability of the model (Table S2). These results reveal the possible practicality of neuralKD.

### Validation of Kawasaki disease predictive model

**Fig. 6:**
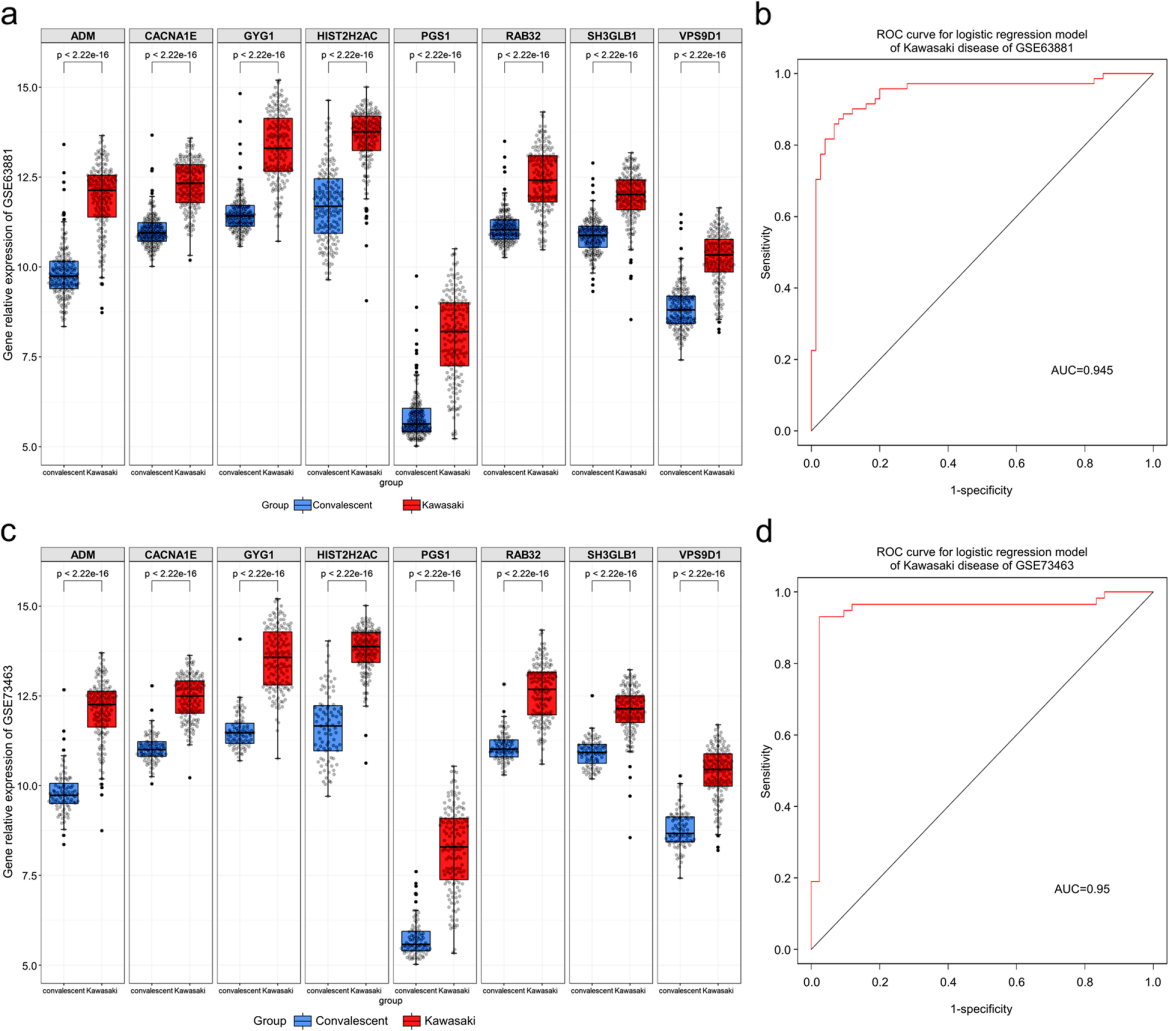
Gene expression validation and performance evaluation of classification model by genes from the neuralKD model. **a** Expression of *VPS9D1*, *CACNA1E*, *SH3GLB1*, *RAB32*, *ADM*, *GYG1*, *PGS1*, and *HIST2H2AC* in GSE63881. Blue: Convalescent group and Red: Kawasaki disease group. **b** AUC verification results with the GSE63881 dataset, which achieved an AUC of 0.945. **c** Expression of *VPS9D1*, *CACNA1E*, *SH3GLB1*, *RAB32*, *ADM*, *GYG1*, *PGS1*, and *HIST2H2AC* in GSE73463. Blue: Convalescent group and Red: Kawasaki disease group. **d** AUC verification results with the GSE73463 dataset, which achieved an AUC of 0.95

To test our hypothesis that neuralKD can predict the progression and prognosis of KD, we introduced two independent KD datasets (GSE63881 and GSE73463). The eight genes were expressed at low levels in the convalescent samples and at high levels in the disease samples (Fig. 6a and c). Moreover, the analysis of the ROC curve showed that the area under the curve (AUC) values corresponding to neuralKD (containing *VPS9D1*, *CACNA1E*, *SH3GLB1*, *RAB32*, *ADM*, *GYG1*, *PGS1*, and *HIST2H2AC*) were 0.945 and 0.95 for the GSE63881 and GSE73463 datasets, respectively (Fig. 6b and d), indicating that neuralKD offers the possibility to predict the progression and prognosis of KD. In addition, we obtained similar findings with GSE109351, which comprised patients with KD, healthy controls, and convalescent samples (Additional File 1, Figure S1). These eight genes were strongly correlated (Additional File 1, Figure S2).

## Discussion

According to our GO enrichment analysis through GSE73461, most DEGs were enriched in neutrophil-related processes, especially degranulation, activation, and differentiation. Additionally, DEGs associated with adaptive immunity responses, such as cytokine-cytokine receptor interaction and Th1/Th2 cell differentiation, were identified using the KEGG pathway enrichment analysis. Our results suggested that KD pathogenesis is closely related to the innate/adaptive immune response, consistent with the findings of previous studies, although the mechanism was not elucidated [28–31]. Moreover, we observed that autoimmune diseases such as systemic lupus erythematosus and inflammatory bowel disease are frequently referenced in KEGG. This suggests that KD exhibits a similar phenotype with autoimmune diseases, characterized by immune system activation of signaling pathways related to IL-1, IL-6, and TNF and the involvement of T/B cells. However, this claim is debatable [32, 33]. Although several DEGs and characteristic pathways were screened, the single bioinformatic analysis method had limited efficacy in identifying candidate genes related to the disease, as further screening was needed when using a large number of DEGs and omitting non-DEGs associated with disease phenotypes. Therefore, we used other techniques, such as WGCNA, RF, and machine learning, to identify the biomarkers associated with KD.

In the present study, we used a subset of data from GSE73461 to select DEGs; hence, the results obtained by artificial grouping according to the clinical phenotype may cause variations. Therefore, we selected all data from GSE73461 using WGCNA, a systems biology method for analyzing molecular interaction mechanisms and resolving correlation networks [34, 35]. Our results revealed characteristic genes in the turquoise module associated with the KD phenotype group. Genes in modules might represent the feature of their corresponding clinical phenotypes by their pattern of expression and, hence, may have possible predictive effects. An increasing number of studies are exploring biomarkers using a combination of WGCNA and DEG identification to ensure the reliability of research [36, 37]. In this study, the combination of DEG screening and WGCNA reduced the number of intersecting genes to select a suitable number of characteristic genes for the subsequent analysis. Furthermore, we noticed that almost all genes in the module were DEGs, supporting the need for further research on DEGs and their related signal pathways, which will facilitate the discovery of new diagnostic indicators and therapeutic targets.

The main difficulty in building classification models using gene expression data is identifying the most meaningful classification indices or features. RF and ANN were used to address this issue in our study based on their high classification accuracy and convenience. Recently, a single algorithm or a combination of these algorithms have been widely used in gene expression data classification, especially disease diagnosis research [38–40]. In this study, we determined characteristic genes related to KD and found several important candidate genes through the RF classifier. Eight genes were then identified using an ANN model and cross-validation. To further validate the neuralKD performance in disease classification, we employed various classification-based methods such as discriminant analysis, PCA, and logistic regression using GSE68004 data. Our results showed that neuralKD exhibited excellent diagnostic performance when validated against multiple machine learning methods except for PCA. Given that PCA is an unsupervised algorithm [41], applied among machine learning methods in our study, and the data were not properly parameterized, the classification efficacy of GSE68004 was low. Recently, the development of machine learning algorithms and availability of gene expression data or clinical data from patients with KD has provided approaches to infer biomarkers for disease diagnosis [42, 43].

These previous studies have established different models for diagnosing KD through either single nucleotide polymorphism or laboratory indicators, demonstrating the value of in-depth research on the molecular mechanism of the disease. In particular, neuralKD obtained in our study showed excellent classification ability of GSE73463 and GSE63881 datasets and the expression levels of the eight genes involved in the model were good indicators of KD prognosis.

Among the eight genes (*VPS9D1*, *CACNA1E*, *SH3GLB1*, *RAB32*, *ADM*, *GYG1*, *PGS1*, and *HIST2H2AC*) in neuralKD, *VPS9D1* encodes a VPS9 domain-containing protein with ATP synthase and GTPase activator activities. Its expression increases in sepsis survivors and has a higher burden of missense variants in sepsis survivors [44]. Similarly, the pathogenesis of KD is closely related to the inflammatory response, and our results also showed low expression of *VPS9D1* in the convalescent group, indicating the value of this gene in evaluating disease progression. *CACNA1E* is a member of the voltage-gated calcium channel family, which comprises key transducers of cell surface membrane potential changes into local intracellular calcium transients that initiate different physiological events [45]. A previous study indicated that the As_2_O_3_-induced inflammatory response depends on Ca overload in chicken myocardial damage [46]. Based on the available information and our study results, we hypothesized that *CACNA1E* is differentially expressed during an inflammatory response, thereby affecting the serious outcomes of KD such as CALs and even CAA dilation, stenosis, thrombosis, and myocardial infarction. Another study using GEO data identified *SH3GLB1*, *PGS1*, and *RAB31* as diagnostic markers for pediatric sepsis, a possible risk factor for KD pathogenesis [47]. Although these risk factors may contribute to the pathogenesis of KD, the bioinformatic analysis based on pediatric sepsis data failed to reveal a direct association with KD. In contrast, our results showed that the eight genes in neuralKD have robust relationships, suggesting a potential mechanism of their interactions in KD. The expression of *ADM*, also called adrenomedullin and associated with coronary artery vasodilation, was downregulated in both healthy individuals and convalescent-phase patients, compared with that in patients with acute KD. This finding is consistent with the results of several previous studies, indicating that *ADM* expression plays a decisive role in the diagnosis and prognosis of KD [48–50]. Importantly, we are committed to conducting in-depth research in the future on the eight genes sourced from neuralKD to verify their effects through in vitro experiments.

## Conclusions

We employed machine learning methods to construct and validate diagnostic models (neuralKD) through multiple datasets using a combination of DEGs screening and WGCNA, resulting in the identification of molecules expected to serve as new biomarkers or therapeutic targets in the future. However, the present study had some limitations. First, conclusions from bioinformatic analyses require further experimental verification. Other unknown genes related to the "core genes" from neuralKD may also play a certain auxiliary role in the pathogenesis and progression of KD, which requires further exploration. Second, due to the large differences in information provided by different GEO datasets, clinical information of samples was omitted when constructing the diagnostic model. Using such varying clinical data may interfere with our analysis and validation results. Third, prospective studies must be conducted to validate the utility of this new model using larger samples.

## Supplementary Information

**Additional file 1:** **Figure S1.** Gene expression validation of classification model by genes from neuralKD model in GSE109351. **(a)** Heatmap of *VPS9D1*, *CACNA1E*, *SH3GLB1*, *RAB32*, *ADM*, *GYG1*, *PGS1*, and *HIST2H2AC*. The correlation between color and relative expression level is displayed in the upper right corner. **(b)** Gene expression of *VPS9D1*, *CACNA1E*, *SH3GLB1*, *RAB32*, *ADM*, *GYG1*, *PGS1*, and *HIST2H2AC* in GSE109351. Yellow: Control group; Blue: Convalescent group and Red: Kawasaki disease group. In (a), the genes with upregulated expression are indicated with red, whereas those with downregulated expression are indicated with blue. **Figure S2.** Correlations of *VPS9D1*, *CACNA1E*, *SH3GLB1*, *RAB32*, *ADM*, *GYG1*, *PGS1*, and *HIST2H2AC* expression in GSE73461 dataset. Red indicates a positive correlation between genes, whereas blue indicates a negative correlation. Each sector in the figure represents the proportion of its correlation. **Table S1.** Performance measure metrics for evaluating the ability of neuralKD on other algorithms. **Table S2**. Ten-time five-fold cross-validation results of AUC from multiple algorithms.

## Data Availability

The gene microarray datasets used in this study, GSE73461, GSE68004, GSE73463, GSE63881, and GSE109351, can be found in the GEO database. GSE73461 can be downloaded from https://www.ncbi.nlm.nih.gov/geo/query/acc.cgi?acc=GSE73461, GSE73463 can be download from https://www.ncbi.nlm.nih.gov/geo/query/acc.cgi?acc=GSE73463, GSE68004 can be download from https://www.ncbi.nlm.nih.gov/geo/query/acc.cgi?acc=GSE68004, GSE63881 can be download from https://www.ncbi.nlm.nih.gov/geo/query/acc.cgi?acc=GSE63881, GSE109351 can be download from https://www.ncbi.nlm.nih.gov/geo/query/acc.cgi?acc=GSE109351.

## Abbreviations

ANN: Artificial neural network
AUC: Area under the curve
CAA: Coronary artery aneurysm
CAL: Coronary artery lesions
DEG: Differentially expressed gene
GEO: Gene expression omnibus
GO: Gene ontology
KD: Kawasaki disease
KEGG: Kyoto encyclopedia of genes and genomes
MDA: Mixed discriminant analysis
PCA: Principal component analysis
QDA: Quadratic discriminant analysis
RF: Random forest
ROC: Receiver operating characteristic
WGCNA: Weighted gene co-expression network analysis
