# Supplementary material for: Diagnostic model based on bioinformatics and machine learning to distinguish Kawasaki disease using multiple datasets
Source: BMC Pediatr. 2022 Aug 30;22:512. doi: 10.1186/s12887-022-03557-y (PMC9425821; doi:10.1186/s12887-022-03557-y)
Supplement: Supplementary file 1 — Additional file 1: Figure S1. Gene expression validation of classification model by genes from neuralKD model in GSE109351. (a) Heatmap of VPS9D1, CACNA1E, SH3GLB1, RAB32, ADM, GYG1, PGS1, and HIST2H2AC. The correlation between color and relative expression level is displayed in the upper right corner. (b) Gene expression of VPS9D1, CACNA1E, SH3GLB1, RAB32, ADM, GYG1, PGS1, and HIST2H2AC in GSE109351. Yellow: Control group; Blue: Convalescent group and Red: Kawasaki disease group. In (a), the genes with upregulated expression are indicated with red, whereas those with downregulated expression are indicated with blue. Figure S2. Correlations of VPS9D1, CACNA1E, SH3GLB1, RAB32, ADM, GYG1, PGS1, and HIST2H2AC expression in GSE73461 dataset. Red indicates a positive correlation between genes, whereas blue indicates a negative correlation. Each sector in the figure represents the proportion of its correlation. Table S1. Performance measure metrics for evaluating the ability of neuralKD on other algorithms. Table S2. Ten-time five-fold cross-validation results of AUC from multiple algorithms. [file 12887_2022_3557_MOESM1_ESM.docx]

***Supplementary appendix***

**Diagnostic model based on bioinformatics and machine learning to distinguish Kawasaki disease using multiple datasets**

Mengyi Zhang^1,2^, Bocuo Ke^1,2^, Huichuan Zhuo^1,2^, Binhan Guo^1,2^*

^1^Department of Laboratory Medicine, West China Second University Hospital, Sichuan University, Chengdu, China

^2^Key Laboratory of Birth Defects and Related Diseases of Women and Children (Sichuan University), Ministry of Education, Chengdu, China

***Corresponding author**: Binhan Guo

Department of Laboratory Medicine, West China Second University Hospital, Sichuan University

No. 20, Section 3, Renmin South Road, Chengdu, Sichuan Province 610041, PR China

Phone: 18081939857

Email: 307408782@qq.com


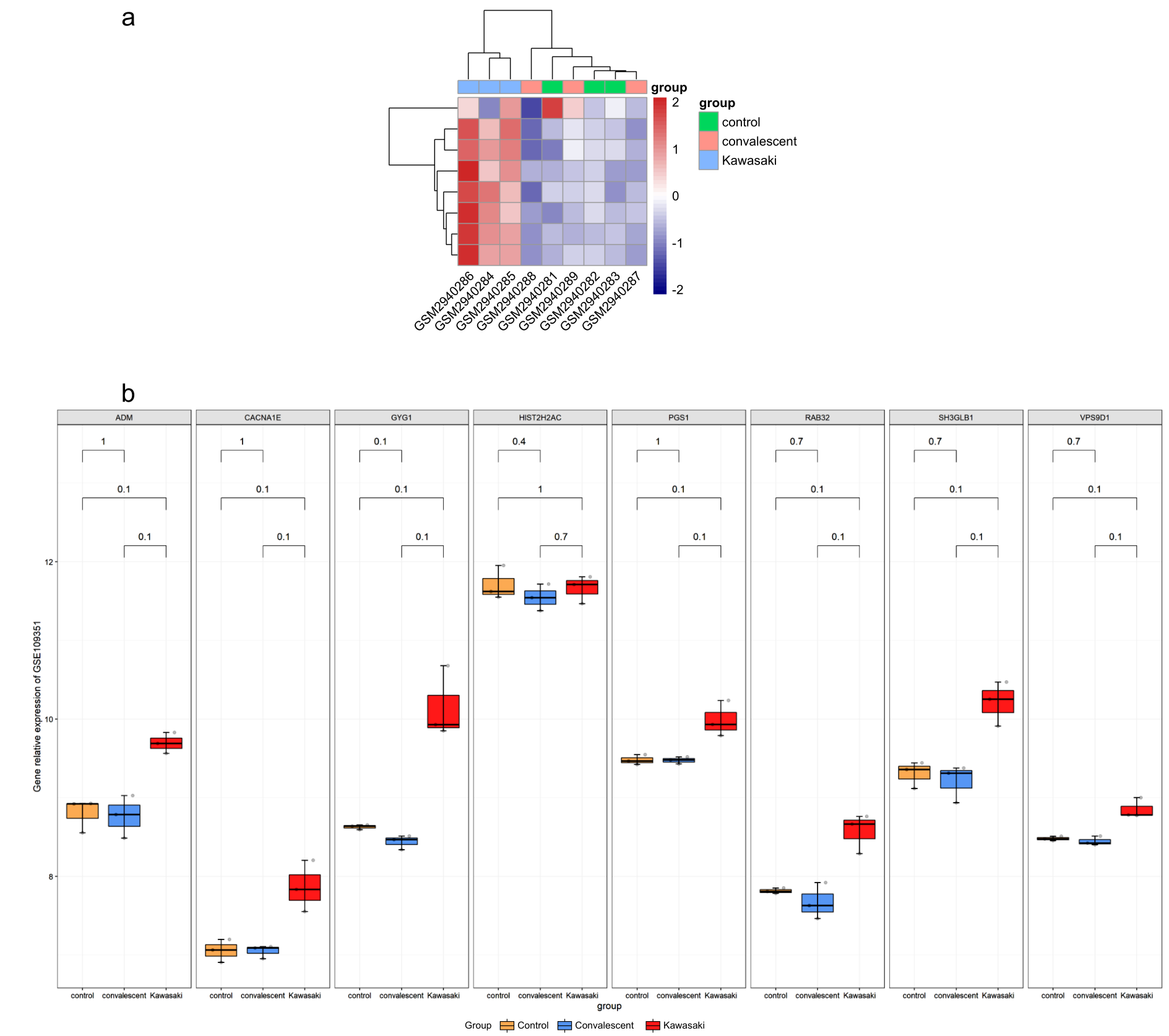


**Figure S1**. Gene expression validation of classification model by genes from neuralKD model in GSE109351. **(a)** Heatmap of *VPS9D1*, *CACNA1E*, *SH3GLB1*, *RAB32*, *ADM*, *GYG1*, *PGS1*, and *HIST2H2AC*. The correlation between color and relative expression level is displayed in the upper right corner. **(b)** Gene expression of *VPS9D1*, *CACNA1E*, *SH3GLB1*, *RAB32*, *ADM*, *GYG1*, *PGS1*, and *HIST2H2AC* in GSE109351. Yellow: Control group; Blue: Convalescent group and Red: Kawasaki disease group. In (a), the genes with upregulated expression are indicated with red, whereas those with downregulated expression are indicated with blue.


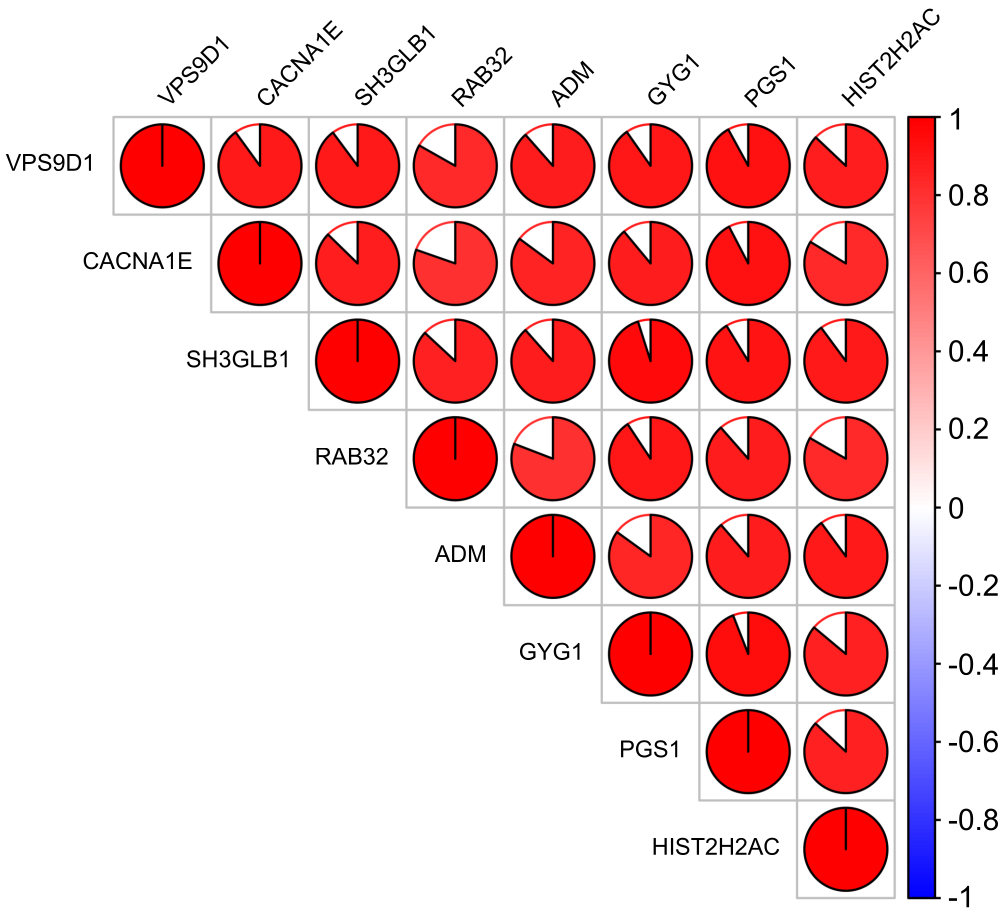


**Figure S2**. Correlations of *VPS9D1*, *CACNA1E*, *SH3GLB1*, *RAB32*, *ADM*, *GYG1*, *PGS1*, and *HIST2H2AC* expression in GSE73461 dataset. Red indicates a positive correlation between genes, whereas blue indicates a negative correlation. Each sector in the figure represents the proportion of its correlation.

**Table S1.** Performance measure metrics for evaluating the ability of neuralKD on other algorithms.

| **Term** | **Accuracy** | **F1 Score** | **AUC** |
| --- | --- | --- | --- |
| neuralKD | 0.912 | 0.938 | 0.865 |
| QDA (training set of GSE68004) | 0.944 | 0.958 | 0.942 |
| QDA (validation set of GSE68004) | 1 | 1 | 1 |
| MDA (training set of  GSE68004) | 0.956 | 0.967 | 0.95 |
| MDA (validation set of GSE68004) | 1 | 1 | 1 |
| logistic model (validation of GSE68004) | 1 | 1 | 1 |
| Validation on GSE63881 | 0.949 | 0.962 | 0.945 |
| Validation on GSE73463 | 0.951 | 0.968 | 0.950 |

**Table S2.** Ten-time five-fold cross-validation results of AUC from multiple algorithms.

|  | **AUC of neuralKD** | **AUC of QDA (training set of GSE68004)** | **AUC of QDA (validation set of GSE68004)** | **AUC of MDA (training set of GSE68004)** | **AUC of MDA (validation set of GSE68004)** | **AUC of logistic model (validation of GSE68004)** | **AUC of Validation on GSE63881** | **AUC of Validation on GSE63881** |
| --- | --- | --- | --- | --- | --- | --- | --- | --- |
| FoldValidation 1 | 0.861 | 0.950 | 1 | 0.951 | 1 | 1 | 0.942 | 0.951 |
| FoldValidation 2 | 0.877 | 0.934 | 1 | 0.938 | 1 | 1 | 0.939 | 0.941 |
| FoldValidation 3 | 0.866 | 0.931 | 1 | 0.965 | 1 | 1 | 0.946 | 0.959 |
| FoldValidation 4 | 0.870 | 0.941 | 1 | 0.949 | 1 | 1 | 0.953 | 0.942 |
| FoldValidation 5 | 0.860 | 0.951 | 1 | 0.961 | 1 | 1 | 0.946 | 0.942 |
| FoldValidation 6 | 0.852 | 0.941 | 1 | 0.941 | 1 | 1 | 0.947 | 0.958 |
| FoldValidation 7 | 0.852 | 0.953 | 1 | 0.951 | 1 | 1 | 0.948 | 0.950 |
| FoldValidation 8 | 0.870 | 0.932 | 1 | 0.962 | 1 | 1 | 0.946 | 0.947 |
| FoldValidation 9 | 0.875 | 0.953 | 1 | 0.937 | 1 | 1 | 0.945 | 0.958 |
| FoldValidation 10 | 0.865 | 0.931 | 1 | 0.964 | 1 | 1 | 0.935 | 0.941 |
